# Oligodendrocyte involvement in Gulf War Illness

**DOI:** 10.1002/glia.23668

**Published:** 2019-07-24

**Authors:** Jillian Belgrad, Dipankar J. Dutta, Samantha Bromley‐Coolidge, Kimberly A. Kelly, Lindsay T. Michalovicz, Kimberly A. Sullivan, James P. O'Callaghan, Richard. Douglas Fields

**Affiliations:** ^1^ Section on Nervous System Development and Plasticity, The Eunice Kennedy Shriver National Institute of Child Health and Human Development (NICHD) National Institutes of Health (NIH) Bethesda Maryland; ^2^ The Henry M. Jackson Foundation for the Advancement of Military Medicine, Inc. Bethesda Maryland; ^3^ Department of Environmental Health Boston University School of Public Health Boston Massachusetts; ^4^ Centers for Disease Control and Prevention Morgantown West Virginia

**Keywords:** acetylcholine, activity‐dependent myelination, cholinergic, corticosterone, Gulf War illness, myelin, organophosphate, plasticity, white matter

## Abstract

Low level sarin nerve gas and other anti‐cholinesterase agents have been implicated in Gulf War illness (GWI), a chronic multi‐symptom disorder characterized by cognitive, pain and fatigue symptoms that continues to afflict roughly 32% of veterans from the 1990–1991 Gulf War. How disrupting cholinergic synaptic transmission could produce chronic illness is unclear, but recent research indicates that acetylcholine also mediates communication between axons and oligodendrocytes. Here we investigated the hypothesis that oligodendrocyte development is disrupted by Gulf War agents, by experiments using the sarin‐surrogate acetylcholinesterase inhibitor, diisopropyl fluorophosphate (DFP). The effects of corticosterone, which is used in some GWI animal models, were also investigated. The data show that DFP decreased both the number of mature and dividing oligodendrocytes in the rat prefrontal cortex (PFC), but differences were found between PFC and corpus callosum. The differences seen between the PFC and corpus callosum likely reflect the higher percentage of proliferating oligodendroglia in the adult PFC. In cell culture, DFP also decreased oligodendrocyte survival through a non‐cholinergic mechanism. Corticosterone promoted maturation of oligodendrocytes, and when used in combination with DFP it had protective effects by increasing the pool of mature oligodendrocytes and decreasing proliferation. Cell culture studies indicate direct effects of both DFP and corticosterone on OPCs, and by comparison with in vivo results, we conclude that in addition to direct effects, systemic effects and interruption of neuron–glia interactions contribute to the detrimental effects of GW agents on oligodendrocytes. Our results demonstrate that oligodendrocytes are an important component of the pathophysiology of GWI.

## Main points

- Oligodendrocytes are important contributors to the pathophysiology of Gulf War illness, a chronic condition with wide‐ranging neurological symptoms.

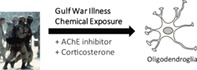

## 1 INTRODUCTION

Gulf War Illness (GWI) is a chronic multi‐symptom disorder that continues to afflict about a third of veterans who returned from the 1990–91 Gulf War (GW), a multi‐nation coalition led by the U.S. against the Iraqi invasion of Kuwait (Steele, Lockridge, Gerkovich, Cook, & Sastre, 2015; White et al., 2016). The cause of GWI is unknown, but exposure to low‐level sarin nerve gas, pesticides and pyridostigmine bromide (PB), all acetylcholinesterase (AChE) inhibitors, have been linked to the etiology of GWI (Sullivan et al., 2018; White et al., 2016). PB, a reversible AChE inhibitor, was ingested by GW veterans as a prophylactic against potential exposure to sarin nerve gas, an irreversible and lethal AChE inhibitor (Sullivan et al., 2003; White et al., 2016). Pesticides, predominantly organophosphates like chlorpyrifos and dichlorvos, were applied, often in excess, to protect against insects in the battle lines along the Mesopotamian marshes (Sullivan et al., 2018). Naturally, the primary focus of GWI research has been on the consequences of disrupting cholinergic synaptic neurotransmission. However, it is unknown how transient disruption of cholinergic synaptic neurotransmission can lead to chronic neurological deficits that have persisted for over two decades in GWI patients. Recent research has shown that neurotransmitters are also involved in communication between axons and myelinating glia. Here, we test the hypothesis that disrupting cholinergic neuro‐glial communication via AChE inhibitors can impair oligodendrocyte development and function, and thereby contribute to the chronic pathophysiology of GWI.

In addition to synaptic release, neurotransmitters are released non‐synaptically along axons through exocytosis at axonal varicosities (Wake et al., 2015) and through ion channels (Fields, 2011; Vizi & Lendvai, 2008). Recent research indicates that oligodendrocyte development and myelination are impaired when this axo‐glial signaling is disrupted. For example, blocking vesicular release of the neurotransmitter glutamate from axons by Botulinum toxin treatment, inhibits local protein synthesis of the major protein in myelin, myelin basic protein (MBP), and impairs induction of myelination on electrically active axons (Wake, Lee, & Fields, 2011). Activity‐dependent myelination has been primarily studied in the context of glutamatergic signaling (Fields, 2015; Kukley, Capetillo‐Zarate, & Dietrich, 2007; Wake et al., 2011; Wake et al., 2015), but the neurotransmitter acetylcholine (ACh) has recently been suggested to influence oligodendrocyte progenitor cell (OPC) proliferation, differentiation, and myelination (De Angelis, Bernardo, Magnaghi, Minghetti, & Tata, 2012; Fields, Dutta, Belgrad, & Robnett, 2017). In the CNS, cholinergic neurons in the basal forebrain extend long‐range axons to broadly innervate the entire cerebral cortex (Luchicchi, Bloem, Viaña, Mansvelder, & Role, 2014; Wu, Williams, & Nathans, 2014). Associated with arousal, focus, and emotional salience, ACh signaling has been found to modulate plasticity of synapses across brain regions including the hippocampus, hypothalamus, and visual cortex (Luchicchi et al., 2014; Picciotto, Higley, & Mineur, 2012). Receptors in cholinergic signaling include the ionotropic nicotinic acetylcholine receptors, and the G‐protein coupled muscarinic receptors. The acetylcholinesterase enzyme (AChE) degrades ACh at cholinergic synapses to terminate synaptic transmission and thereby prevents neuronal hyperactivity and excitotoxicity. In contrast to synaptic transmission, far less is known about the effects of acetylcholine and AChE inhibitors on oligodendrocyte development and function.

The severity of GWI correlates with exposure to pesticides and PB in a dose‐dependent manner (Steele, Sastre, Gerkovich, & Cook, 2012; Wolfe, Proctor, Erickson, & Hu, 2002). Butyrylcholinesterase (BChE) is a nonspecific cholinesterase that hydrolyzes choline‐based esters including toxicants such as organophosphate pesticides. A genetic variant of BChE, that encodes a less active form of BChE and hence is less adept at neutralizing GW toxicants, is one of the reported genetic risk factors for GWI (Steele et al., 2015). GW veterans with the atypical BChE gene are more susceptible to developing GWI upon exposure to GW pesticides. Together these findings highlight the important role of AChE inhibitors in the pathophysiology of GWI (Golomb, 2008).

Recent studies provide support for this previously unexplored hypothesis of involvement of myelinating glia in the pathophysiology of GWI. Brain imaging studies have reported white matter abnormalities in GW veterans (Chao, Zhang, & Buckley, 2015; Heaton et al., 2007) and such disruption has been associated with the key diagnostic symptoms of GWI: musculoskeletal pain (Rayhan et al., 2013; Van Riper et al., 2017), impaired attention (Janulewicz et al., 2017), disturbances of mood (Van Riper et al., 2017), and chronic fatigue (Rayhan et al., 2013). However, white matter is a complex tissue comprised of axons, astrocytes, oligodendrocytes, vascular cells, and microglia. Therefore, alterations in white matter detected by MRI could result from many types of cellular perturbations, including changes in axon number, diameter, tortuosity, vascular changes, alterations in astrocyte number or morphology, as well as direct effects on myelin. Furthermore, loss of myelin could be secondary to loss of axons, rather than a direct effect on oligodendrocytes.

Myelination proceeds in different brain regions at different times, but the process continues through adolescence into early adulthood. In the prefrontal cortex (PFC), myelination continues during the early 20s (Miller et al., 2012), a demographic accounting for roughly 50% of deployed GW soldiers (Veterans Affairs, 2011). Additionally, there is a reserve pool of proliferative NG2+ cells in the adult brain, which have the potential to generate oligodendrocyte lineage cells throughout life (Nishiyama, Suzuki, & Zhu, 2014; Kang, Fukaya, Yang, Rothstein, & Bergles, 2010). Epidemiological data indicate that GW veterans who report impaired cognition as their prominent symptom were significantly younger than their GW veteran counterparts with no‐symptoms or who experience primarily sensory symptoms (Gopinath et al., 2012). This pattern is consistent with possible involvement of disrupted PFC myelination in GWI and presents a compelling hypothesis for the neurological and cognitive impairments of GWI.

Here we test the hypothesis that acute exposure to AChE inhibitors affects oligodendrocyte proliferation, differentiation, survival, and myelination. These studies were carried out in an established Center for Disease Control (CDC) rat model of GWI (Koo et al., 2018) in combination with studies in cell culture. Key diagnostic features of GWI include musculoskeletal pain, impaired cognitive functioning, disturbances of mood, and debilitating fatigue; symptoms that have persisted over time (Binns et al., 2008; Maule et al., 2018; White et al., 2016). GWI animal models replicate many of these symptoms (Zakirova et al., 2016), including impaired working memory (Phillips & Deshpande, 2018) and social memory (Zakirova et al., 2016). The established animal model of GWI includes treatment with corticosterone (Cort) for 7 days before exposure to diisopropyl fluorophosphate (DFP), an irreversible AChE inhibitor used as a proxy for sarin nerve gas (Koo et al., 2018; O'Callaghan, Kelly, Locker, Miller, & Lasley, 2015; Zakirova et al., 2016). This necessitates studying the effects of Cort exposure independently, and together with DFP, on oligodendrocyte development and function. Interaction between these two agents is possible in influencing oligodendroglial biology. Stress and corticosterone have been shown to influence oligodendrocyte and myelin biology outside of the context of GWI. Corticosterone treatment has been shown to inhibit OPC proliferation (Alonso, 2000), promote OPC differentiation (Mann et al., 2008), and shorten the node of Ranvier length (Miyata et al., 2016). Inhibition of ACh signaling promotes remyelination in experimental autoimmune encephalomyelitis studies, an animal model of Multiple Sclerosis (MS), and in human MS clinical trials (Abiraman et al., 2015; Green et al., 2017; Li, He, Fan, & Sun, 2015; Liu et al., 2016; Mei et al., 2014; Welliver et al., 2018). Although this treatment is therapeutic for a demyelinating disease, disrupting ACh signaling may be detrimental to oligodendroglia in other contexts. In studies reported here, effects of DFP and Cort were investigated in adult rats in the GWI model, in the PFC, which is still undergoing myelination, and in subcortical white matter (corpus callosum), which in comparison to PFC is undergoing less active myelination.

Both DFP and Cort may act directly on oligodendroglia and indirectly by disrupting neuron–glia interactions. In addition to disrupting cholinergic signaling, these agents could have non‐cholinergic actions or produce systemic effects, such as vascular and immune responses, that could have detrimental effects on myelinating glia. These alternatives were investigated using a combination of in vivo and in vitro studies. The results indicate that exposure to DFP, with and without Cort, disrupts oligodendrocyte development in the GWI animal model. In vitro experiments using purified oligodendrocyte lineage cell monocultures, in the absence of detectible ACh, indicate that DFP and corticosterone have direct effects on oligodendroglial cell proliferation and survival, but these effects differ in important respects from those seen in the animal model of GWI. This finding distinguishes the consequences of the systemic and non‐cholinergic effects of GW agents from their role in disrupting cholinergic signaling between axons and oligodendroglia as AChE inhibitors. The results of this study support the conclusion that oligodendrocyte biology is an important contributor to the pathophysiology of GWI and that GW agents impair cholinergic signaling between axons and myelinating glia but also have direct non‐cholinergic effects on these cells. Cort treatment in the GWI animal model has additional and, in some respects, counteracting effects to DFP on oligodendrocytes. The findings suggest possible therapeutic approaches to alleviate the chronic neurological symptoms in GW veterans from exposure to anticholinesterase agents during the GW.

## 2 MATERIALS AND METHODS

### 2.1 Mixed glial cell culture preparation

Primary rat mixed glial cell cultures were generated from P1‐2 day old wild‐type Sprague–Dawley rat pups. Briefly, pups were decapitated, and their cerebral cortices were isolated, minced, separated into a single cell suspension, and plated in T75 flasks. Mixed glial cultures were grown in Dulbecco's Modified Enriched Media (DMEM, ThermoFisher Scientific, Waltham, MA, Cat. No. 11995–065) that contained high glucose, L‐glutamine, phenol red, and sodium pyruvate with 10% Fetal Bovine Serum (FBS, ThermoFisher Scientific, Cat. No. 16000–044) for 3 weeks at 37°C and 10% CO_2_.

### 2.2 Oligodendrocyte progenitor cell purification

At 3–4 weeks post‐dissection, flasks were shaken (180 rpm) for an hour at 37°C to remove microglia and dead cells, followed by a compete media change and an overnight shake under the same conditions. Media was collected from shaken flasks and plated onto two 6 cm tissue culture dishes per flask, for 15 min, to separate OPCs from heavier endothelial and astrocyte cells. Supernatant from 6 cm dishes was collected and centrifuged for 10 min at 1200 rpm. Cells were then plated onto 25 mm glass coverslips coated with 0.1 mg/mL poly‐L‐lysine (PLL) (Sigma‐Aldrich, P9155) and 0.1 mg/mL poly‐L‐ornithine (PLO) (Sigma‐Aldrich, P3655). Coverslips were used for calcium imaging 1–3 days post‐plating. 80–90% of the cells on coverslips used for experiments were oligodendrocytes as confirmed by immunocytochemistry with the pan‐oligodendrocyte marker, Olig2. Purified OPCs were grown in DMEM+10% FBS (described above) without additional growth factors.

### 2.3 Calcium imaging

Calcium imaging was performed on OPCs 2–3 days post‐plating using the fluorescent calcium chelator dye, Fura2 AM (Invitrogen, Carlsbad, CA Cat. No. F1221). Fifty microgram Fura‐2 AM was added to 50 μL Pluronic Acid (Invitrogen, Cat. No. P3000‐MP). Fifteen microliter of the Fura‐2 Pluronic acid solution was added to HEPES buffer and brought to a total volume of 1.5 mL. HEPES buffer (pH 7.4) consisted of 8 g/L Sucrose, 1 g/L D‐glucose, 20 mM HEPES stock (150 nM NaCl, 10 nM HEPES, 3 mM KCl), 2 mM CaCl_2_, 2 mM MgCl_2_. One mililiter of the diluted Fura‐2 was added to a 35 mm dish containing the 25 mm coverslip containing OPCs for 15 min in the dark at 37°C. After 15 mins, 1 mL of HEPES buffer was added and the cells were then incubated a second time for 15 min in the dark at room temperature. Coverslip was washed one time for 10 min with HEPES buffer before use. MetaFluor Software (Molecular Devices) was used to image and measure fluorescence emission at 340 and 380 nm excitation wavelengths. Acetylcholine was diluted in HEPES buffer to 1 and 50 μM concentrations. Intracellular Fura‐2 levels were calibrated using 10 μM A23187 with and without EGTA or Ca^2+^ in buffer. Calcium concentrations were calculated from fluorescence levels using the equation derived by Grynkiewicz, Poenie, and Tsien (1985). While sampling, N was defined as a coverslip while n was defined as a cell.

### 2.4 Oligodendrocyte differentiation

OPCs were differentiated in N1 media with 0.2% FBS. OPCs were plated from flasks, as described above, in DMEM + 10% FBS for 24 hr and then switched to N1 media + 0.2% FBS for the remainder of the experiment.

### 2.5 Astrocyte cultures

At 3–4 weeks post dissection, flasks were shaken overnight at 37°C and 1200 rpm to remove microglia and dead cells. Media was removed, and flasks were washed twice with sterile Earle's Balanced Salt Solution (EBSS). Trypsin, warmed to 37°C, was added to the flasks and trypsinization was stopped 10 min later with 1:1 addition of DMEM + 10% FBS. Cells were collected and centrifuged at 1200 rpm and then plated on PLL/PLO coated 25 mm glass coverslips.

### 2.6 In vitro cell culture treatments

OPCs isolated from postnatal day 2 (P2) cerebral cortex were treated 24 hr after plating. Cells were fixed with 4% paraformaldehyde and immunocytochemistry was performed 72 hr following treatment.

### 2.7 Immunocytochemistry

Coverslips were fixed with 4% paraformaldehyde (Electron Microscopy Sciences, Hatfield, PA) in Phosphate Buffered Saline (PBS, Sigma‐Aldrich, St. Louis, MO) for 20 min followed by the addition of 0.1% Triton X‐100 (Sigma‐Aldrich) in PBS for 5 min. The coverslips were washed three times with PBS and blocked for 1 hr in 5% Goat Serum in PBS (Fisher Scientific). The coverslips were then incubated overnight at 4°C with the primary antibody. The coverslips were then washed three times with PBS and incubated with secondary antibody for 2 hr at room temperature. Coverslips were washed three times and plated on microscope slides with Vectorshield Antifade Mounting Medium with DAPI (Vector Laboratories, Burlingame, CA) for imaging. For immunocytochemistry, the primary antibodies used were: Olig2 (EMD Millipore, Rabbit) at 1:500, Olig2 (EMD Millipore, Mouse, Cat. No. MABN50) at 1:500, Myelin Basic Protein (EMD Millipore, Chicken) at 1:200, AChE (Invitrogen, Mouse) at 1:200, Cleaved Caspase‐3 (Asp175) (Cell Signaling Technology, Rabbit) at 1:200. The secondary antibodies used were: Alexa Fluor 488 (Thermo Fisher Scientific, Goat Anti‐Rabbit IgG) at 1:1000, Alexa Fluor 488 (Thermo Fisher Scientific, Goat Anti‐Mouse) at 1:1000, Alexa Fluor 568 (Thermo Fisher Scientific, Goat Anti‐Mouse) at 1:1000, Alexa Fluor 568 (Thermo Fisher Scientific, Goat Anti‐Rabbit) at 1:1000, Alexa Fluor 555 (Thermo Fisher Scientific, Goat Anti‐Chicken) at 1:1000.

### 2.8 Pharmacological agents

Acetylcholine (Sigma), DFP (Sigma), Corticosterone (Sigma) and Calcium Ionophore A23187 (Sigma) were used in the study. Ethanol (0.6%) was used as the corticosterone vehicle for in vivo and in vitro experiments. Doses used for each experiment are described in the results section.

### 2.9 GWI animal model

Adult male Sprague Dawley rats aged 6–8 weeks received Corticosterone (Cort, 200 mg/L in 0.6% ethanol) in drinking water for days 1–7, followed by a single subcutaneous (s.c.) injection of DFP (1.5 mg/kg) on the morning of day 8. Animals were sacrificed 12, 24, 72 hr, or 21 days after DFP exposure.

### 2.10 Tissue sectioning

Rats were sacrificed by decapitation and brains were rapidly removed. One hemisphere was frozen for protein analysis and the other was post‐fixed in 4% paraformaldehyde overnight and cryopreserved in 30% sucrose for up to 4 days. Following adequate cryopreservation, brains were embedded in optimal cutting temperature (OCT) embedding media (Fisher Healthcare). Embedded tissue was cyrosectioned into 14 μm thick sagittal sections**.**

### 2.11 Immunohistochemistry

Tissue sections were brought to room temperature and then rinsed one time with PBS to dissolve the embedding medium OCT. The sections were then incubated in Citrate buffer, pH 6, at 94°C for 10 min to promote epitope retrieval. The tissues sections were then rinsed three times with PBS containing 0.1% TritonX‐100 and blocked for 1 hr in blocking buffer (5% normal goat serum and 0.1% Triton X‐100 in PBS). Sections were incubated overnight at 4°C with the primary antibodies. Primary antibodies used include: APC/CC1 (Millipore, Mouse) at 1:500; Olig2 (Millipore, Rabbit) at 1:500; Ki67 (Abcam, Rabbit) at 1:500; Olig2, (Millipore, Mouse) at 1:100. The following day, the tissue sections were rinsed three times with PBS containing 0.3% TritonX‐100 and then incubated for 2 hr at room temperature with secondary antibodies (listed in the Immunocytochemistry methods section) at 1:200 dilution. Tissue sections were then rinsed three times with PBS and mounted on coverslips using mounting medium (Vectashield Antifade Mounting Medium with DAPI). Ki67 staining was performed on animals fixed 24 hr after treatment. CC1 staining was performed on animals fixed 21 days after treatment. A fluorescent light microscope with AxioCam MRm was used to acquire 10 images per region per animal at 40 X magnification.

### 2.12 Acetylcholine release assay

Cells were plated onto coverslips as described above and incubated with 150 μL buffer or DFP at 37°C. Conditioned buffer was collected, and flash frozen after 4 hr. Buffer was analyzed with a Choline/Acetylcholine fluorometric assay kit (Abcam). To measure total levels of acetylcholine and choline ([Ach + Ch]), AChE enzyme was used in the buffer. To measure choline levels excluding acetylcholine, ([ACh]), DFP was added to the buffer with no AChE.

### 2.13 Immunoblotting

To extract proteins from tissue and cell culture, samples were lysed in RIPA buffer (Sigma Aldrich) with protease inhibitor cocktails (Complete Mini EDTA‐free Protease Inhibitor Cocktail, Sigma Aldrich). Lysate was mixed with LDS sample buffer (Thermo Fisher Scientific) and electrophoresed in a 4–12% Bis‐Tris Gel (Invitrogen) for 2 hr at 150 V in MOPS‐SDS running buffer (Thermo Fisher Scientific). The samples were transferred to PVDF membrane (Immobilon‐P, Millipore) overnight at 4°C in Tris‐Glycine transfer buffer (Thermo Fisher Scientific). Membranes were blocked in blocking buffer, containing TBS (10 mM Tris–HCl, pH 7.5, 0.9% NaCl), 0.1% (vol/vol) Triton X‐100 and 5% (wt/vol) bovine serum albumin (MP Biomedicals) or 5% nonfat dry milk (American Bio), for 1 hr at room temperature (RT). The appropriate primary antibody was diluted in blocking buffer and incubated overnight with the PVDF membrane at 4°C. Primary antibodies used were: GAPDH (Cell Signaling Tech, Rabbit) used at 1:4000; NSE (Abcam, Rabbit) used at 1:2000; GAPDH (Encor, Mouse) used at 1:4000 dilution; MBP (Millipore, Rabbit) used at 1:1000; GFAP (Invitrogen, Rabbit) used at 1:500, GAP43 (Millipore Sigma Aldrich, Rabbit) used at 1:1000, NG2 (Abcam, Mouse) used at 1:1000. The PVDF membrane was washed four times, 15 min each, in washing buffer, TBS (10 mM Tris–HCl, pH 7.5, 0.9% NaCl) and 0.1% (vol/vol) Triton X‐100. The corresponding secondary antibody, diluted in blocking buffer, was then incubated with the PVDF membrane for 2 hours at RT. Secondary antibodies that were used include ECL Anti‐Mouse IgG Horseradish Peroxidase‐linked F(ab’)2 fragment or ECL Anti‐Rabbit IgG Horseradish Peroxidase‐linked F(ab’)2 fragment. Chemiluminescent substrate was applied for 10 min (SuperSignal West Pico Plus, Thermo Scientific). Membranes were quantified with densitometry using Image J software and normalized to NSE loading control. In reported bar graphs of data, each treatment condition was normalized to saline control levels.

### 2.14 Animal protocol

All animal studies for in vivo experiments were performed under protocols approved by the Institutional Animal Care and Use Committee of the Centers for Disease Control and Prevention, National Institute for Occupational Safety and Health, and the animal facility was certified by AAALAC International. All animal studies for in vitro experiments were performed at the Section on Nervous System Development and Plasticity, Bethesda, MD, according to Animal Study Protocol #15–007, and were approved by the Institutional Animal Care and Use Committee (IACUC), *Eunice Kennedy Shriver* National Institute of Child Health and Human Development (NICHD), National Institutes of Health (NIH). All animals for in vivo and in vitro studies were approved through the Department of Defense Animal Care and Use Review Office (ACURO).

### 2.15 Statistics

Statistics were performed with Minitab 18 (Minitab Inc., State College, PA) and GraphPad Prism 7 (GraphPad Software Inc., La Jolla, CA). Immunoblot data were analyzed with One‐Way ANOVA (Analysis of Variance) followed by Dunnett's multiple comparison post‐test. Cell counting data from histology and cell‐culture experiments were analyzed using the Chi‐Squared statistical test. Figures were made in SigmaPlot 14.0 (Systat Software Inc., San Jose, CA). While sampling for immunoblotting, N was defined as an animal. For histology, *N* was defined as an animal and *n* was defined as a microscopic field of view. For in vitro imaging, *N* was defined as biological experimental replicates and n was defined as a microscopic field of view. For calcium imaging, *N* was defined as a coverslip and *n* was defined as an individual cell. Grouped data are mean ± *SEM* unless stated otherwise.

## 3 RESULTS

### 3.1 OPCs engage in cholinergic signaling

**Figure 1:**
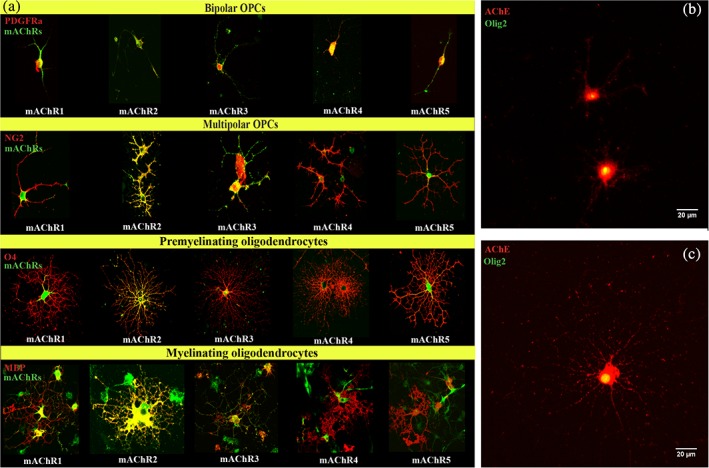
Expression of muscarinic receptors and acetylcholinesterase in oligodendrocyte lineage cells. (a) Expression of muscarinic acetylcholine receptors (mAChRs) 1–5 at various stages of oligodendrocyte development, from immature bipolar progenitors to highly branched mature oligodendrocytes, in primary cultures of oligodendrocyte lineage cells. Red are oligodendrocyte markers; green are muscarinic ACh receptors. (b) AChE expression in immature OPCs. Red is AChE; green is Olig2. (c) AChE expression in mature oligodendrocytes. Red is AChE; green is Olig2

To understand the etiology of white matter disruption present in veterans with GWI, we investigated how Cort exposure and disrupting cholinergic signaling through AChE inhibition may interfere with oligodendrocyte biology. To do so, we first determined the capacity for OPCs to engage in cholinergic signaling across development. Immunocytochemistry indicated that all cells of the oligodendrocyte lineage express muscarinic acetylcholine receptors (mAChRs) M1–M5 in vitro and expression of these receptors varied in abundance and localization across oligodendrocyte development from immature progenitors to mature myelinating cells (Figure 1a, Table 1). Similarly, AChE was expressed on the membrane of OPCs grown for 24 hr in growth medium (Figure 1b) and oligodendrocytes grown for 3 days in differentiation medium (Figure 1c). The function of AChE in oligodendrocytes is unclear (Fields et al., 2017), but sarin gas and other AChE inhibitors would act on both neuronal and glial AChEs.

**Table 1:** Expression of mAChRs on oligodendrocyte lineage cells

Stages of the oligodendrocyte lineage	Cellular compartments	Muscarinic receptors				
		mAChR1	mAChR2	mAChR3	mAChR4	mAChR5
**Bipolar OPCs (PDGFRa+)**	Cell body	**+++**	**+++**	**++**	**+**	**+++**
	Cell processes	**+++**	**+**	**++**	**‐**	**++**
**Multipolar OPCs (NG2+)**	Cell body	**+++**	**+++**	**++**	**+**	**+++**
	Cell processes	**+**	**+++**	**++**	**‐**	**++**
**Premyelinating OLs (O4+)**	Cell body	**+++**	**+++**	**++**	**+**	**+++**
	Cell processes	**+**	**+++**	**++**	**‐**	**++**
**Mature OLs (MBP+)**	Cell body	**+++**	**+++**	**++**	**+**	**++**
	Cell processes	**+**	**+++**	**++**	**‐**	**+**

*Note*: Plus (+) sign indicates relative qualitative levels of receptor expression; dash (−) sign indicates no observable expression.

**Figure 2:**
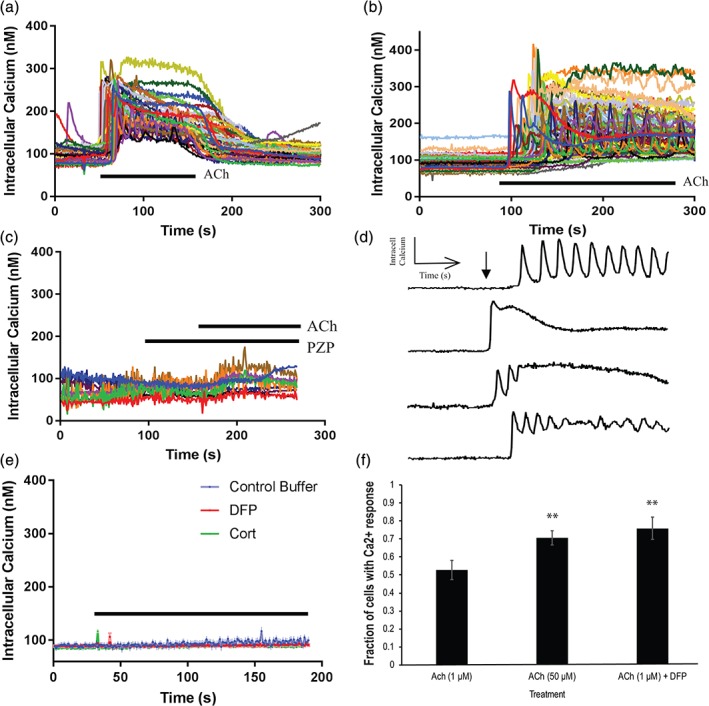
ACh induces OPC intracellular calcium response. (a) 50 μM ACh (*N* = 5, *n* = 56). Line indicates duration of treatment. (b) 1 μM ACh (*N* = 5, *n* = 91). Line indicates duration of treatment. (c) Inhibition with mAChR M1 inhibitor, Pirenzepine (PZP, 50 μM), to confirm specificity of mAChR induced calcium response (*N* = 3, *n* = 9). Line indicates duration of treatment. PZP pretreatment onset at 40 s and co‐treatment of PZP with 50 μM ACh beginning at 160 s. (d) Representative waveform traces of data presented in b. Arrow indicates onset of ACh treatment. (e) DFP (50 μm) alone and Cort (5 μM) alone had no effect on intracellular calcium mobilization in OPCs. (DFP: *N* = 4, *n* = 95; Cort: *N* = 3, *n* = 36). (f) DFP pretreatment followed by ACh significantly increased the fraction of cells that respond to ACh (52.8 ± 5.37% vs. 75.9 ± 6.21%, *t*[15 dishes] = 2.131, *p* = 0.005). Fifty micromolar ACh significantly increased the number of cells that respond to ACh compared to 1 μM ACh (52.8 ± 5.37% vs. 70.6 ± 3.92%, *t*[15 dishes] = 2.947, *p* = 0.0118). Student's *t*‐test was performed comparing ACh (1 μM) and ACh (1 μM) vs. DFP. * indicates *p* < 0.05; ** indicates *p* < 0.01

We performed live‐cell calcium imaging to determine if the mAChRs expressed on OPCs were functional. M1, M3, and M5 receptors signal via activating intracellular Ca^2+^, but M2 and M4 receptors signal through cAMP. Live‐cell calcium imaging of OPC monoculture demonstrated that OPCs respond to 50 μM ACh (Figure 2a, *N* = 5, *n* = 56) and to concentrations as low as 1 μM ACh, (Figure 2b, *N* = 5, *n* = 91) with robust and heterogenous calcium kinetics indicating mAChRs were functional on OPCs. ACh doses of 50 and 1 μM were chosen because similar ACh concentrations have been used previously in studies of the cholinergic neuromuscular synapse (Vianney, Miller, & Spitsbergen, 2014). The response was mediated by mAChR activation, as pretreatment with the m1AChR inhibitor, Pirenzepine (50 μM), eliminated intracellular ACh‐mediated Ca^2+^ signaling (Figure 2c, *N* = 3, *n* = 9). The dynamics of intracellular calcium responses varied in different cells in response to ACh (1 μM). The responses included prolonged oscillations, dampened oscillations, and a sharp rise to peak which plateaued and partially recovered (Figure 2d). These varied waveforms suggest multiple underlying intracellular Ca^2+^ release, extrusion, and sequestration processes, as well as heterogeneity in mAChR type and expression levels, in the cell population stimulated with ACh.

The calcium imaging experiments were performed in HEPES Buffer (ingredients defined in Materials and Methods), which does not contain ACh. DFP treatment alone (*N* = 4, *n* = 95) or Cort alone (*N* = 3, *n* = 36) had no effect on intracellular calcium levels measured over a 300 s treatment (Figure 2e). Thus, DFP and Cort do not influence intracellular Ca^2+^ signaling in OPCs in the absence of ACh. No ACh was detected in buffer conditioned by astrocyte or OPCs for 4 hr using an acetylcholine fluorometric assay with a threshold sensitivity of 100 pmol (*N* = 3). The lack of measurable ACh in OPC and astrocyte monocultures suggests that ACh is not secreted from either OPCs or astrocytes. This supports the hypothesis that release of ACh by neurons signal to oligodendrocytes and that disruption of this neuro‐glial signaling could disrupt oligodendroglial development and function.

We therefore applied low concentrations of ACh to OPC cultures, together with DFP, to test whether inhibiting AChE activity on oligodendrocytes would alter their Ca^2+^ responses. The results showed that DFP treatment in the presence of 1 μM ACh increased the percentage of cells that responded to ACh (Figure 2f, 52.8 ± 5.37% vs. 75.9 ± 6.21%, *t*[15 dishes] = 2.131, *p* = 0.005). This suggests that inhibition of AChE on OPC cell membrane by DFP increased the concentration of ACh in the extracellular environment, thus eliciting responses from more OPCs. A larger percentage of cells also responded to 50 μM ACh compared with the 1 μM treatment (Figure 2f, 52.8 ± 5.37% vs. 70.6 ± 3.92%, *t*[15 dishes] = 2.947, *p* = 0.0118; 50 μM: *N* = 5, *n* = 101). We conclude that AChE expressed on OPCs is functional and that its inhibition in the presence of ACh leads to increased ACh‐dependent Ca^2+^ signaling, which could influence OPC development and function.

### 3.2 Direct effects of GW agents on oligodendrocyte biology

Any effect on oligodendrocyte development and function in the GWI animal model or in GW veterans could be due to direct effects on biology of oligodendrocyte lineage cells, or due to interruption of cholinergic neuron‐oligodendrocyte communication or caused by indirect systemic effects resulting from exposure to Cort and DFP. Cholinergic signaling and stress hormone signaling have diverse biological effects in the CNS, PNS, the cardiovascular, and the immune system. Therefore, to test for direct, non‐systemic effects of the GW agents on OPCs, we exposed OPCs in vitro to the AChE inhibitor, DFP, and to Cort. We performed a dose response study with DFP (Figure S1a) and Cort (Figure S1b) to determine the most appropriate treatment concentration to avoid toxicity (defined by Olig2+ cell counts). Based on the dose response curves, we adopted an OPC treatment paradigm using 1 μM Cort, 1 μM DFP, and a combined Cort+DFP condition, as used in the GWI animal model. OPCs in growth medium were treated 24 hr after plating and were examined via immunocytochemistry 72 hr following treatment.

**Figure 3:**
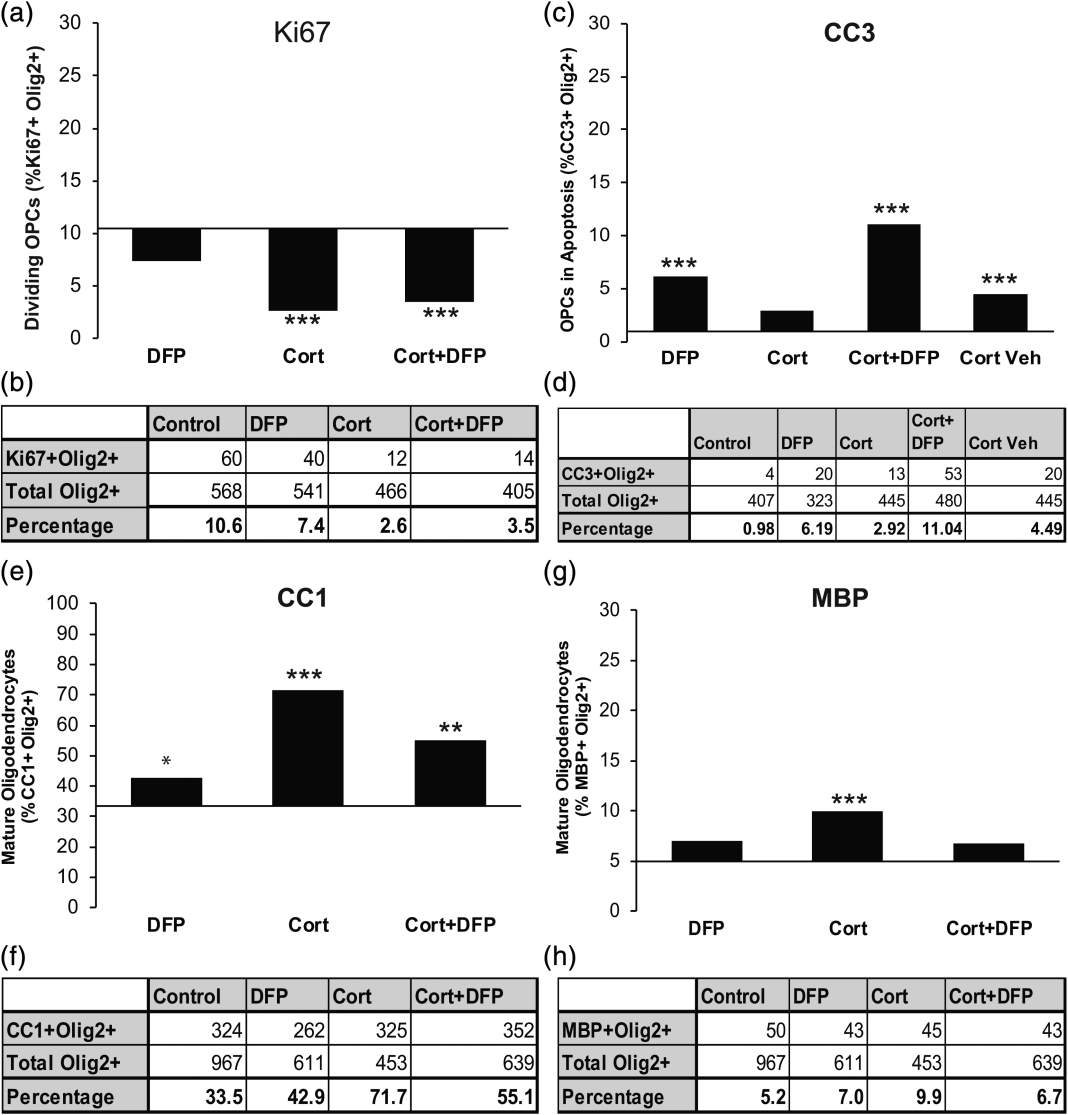
In vitro exposure to GW agents disrupts OPC development. (a) Immunocytochemical staining of OPC monoculture using Ki67 and Olig2 primary antibodies. Cort (χ^2^ [1, *n* = 466] = 26.604, *p* < 0.001) and Cort+DFP (χ^2^ [1, *n* = 405] = 11.150, *p* = 0.001) significantly reduced OPC proliferation, identified as Ki67+ Olig2+ cells, compared to Cort condition. DFP alone had no effect on oligodendrocyte proliferation (χ^2^ [1, *n* = 541] = 2.436, *p* = 0.119). Baseline indicates control value. (b) Table of raw Ki67+ Olig2+ cell counts used to generate part (a). (c) Immunocytochemical staining of OPC monoculture using cleaved caspase 3 (CC3) and Olig2 primary antibodies. DFP (χ^2^ [1, *n* = 323] = 14.316, *p* < 0.001) and Cort+DFP (χ^2^ [1, *n* = 480] = 11.663, *p* = 0.001) treatments significantly increased OPC apoptosis, identified as cleaved caspase 3+ cells. Cort had no effect on apoptosis (χ^2^ [1, *n* = 445] = 1.432, *p* = 0.23). Baseline indicates control value. (d) Table of CC3+ Olig2+ cell counts were used to generate percentages in part (c). (e) Immunocytochemistry staining of oligodendrocyte monoculture (grown in differentiation media for 5 days total) using CC1 and Olig2 primary antibodies. DFP alone, (χ^2^ [1, *n* = 611] = 6.371, *p* = 0.012), Cort alone (χ^2^ [1, *n* = 453] = 28.962, *p* < 0.001) and Cort+DFP (χ^2^ [1, *n* = 639] = 11.133, *p* = 0.001) increased CC1+ mature oligodendrocytes. Baseline indicates control value. (f) Table of CC1+ Olig2+ cell counts used to generate percentages in part (e). (g) Immunocytochemical staining of oligodendrocyte monoculture (grown in differentiation media for 5 days total) using MBP and Olig2 primary antibodies. Cort alone increased MBP+ mature oligodendrocytes (χ^2^ [1, *n* = 453] = 8.419, *p* = 0.004). DFP (χ^2^ [1, *n* = 611] = 2.083, *p* = 0.149) and Cort+DFP (χ^2^ [1, *n* = 639] = 2.449, *p* = 0.118) had no significant effect on counts of MBP+ mature oligodendrocytes. Baseline indicates control value. (h) Table of MBP+ Olig2+ cell counts used to generate percentages in part (g). * indicates *p* < 0.05; ** indicates *p* < 0.01, *** indicates *p* < 0.001. Significance is determined by comparing all treatment conditions with the control condition. DMEM + 10% FBS (used in experiments for parts a–d) or differentiation media (used in experiments for parts e–h). DFP values were compared to control. Cort and Cort+DFP values were compared relative to the “Cort vehicle” control

The data showed that Cort treatment significantly decreased OPC proliferation (Ki67+ Olig2+ cells) (Figure 3a, 10.6 vs. 2.6% respectively, χ^2^[1, *n* = 466] = 26.604, *p* < 0.001). DFP alone had no effect on OPC proliferation (Figure 3a, 10.6 vs. 7.4%, respectively, χ^2^[1, *n* = 541] = 2.436, *p* = 0.119). Cort+DFP co‐treatment, as used in the GWI animal model, also decreased OPC proliferation due to the effect of Cort (Figure 3a, 10.6 vs. 3.5%, respectively, χ^2^[1, *n* = 405] = 11.150, *p* = 0.001). Cell counts used to determine Ki67+ Olig2+ frequency are reported in Figure 3b.

Apoptosis, identified by cleaved caspase 3 (CC3) expression, was increased by DFP treatment (Figure 3c, 0.98 vs. 6.19%, respectively, χ^2 ^[1, *n* = 323] = 14.316, *p* < 0.001) and by Cort+DFP (Figure 3c, 0.95 vs. 11.04%, respectively, χ^2^ [1, *n* = 480] = 11.663, *p* = 0.001) treatment due to the effect of DFP. The vehicle used to dissolve Cort, (0.00004% ethanol) caused a small, but statistically significant increase in apoptosis when used alone (Figure 3c, 0.98 vs. 4.49% respectively, χ^2^[1, *n* = 480] = 37.06, *p* < 0.001), but in combination with Cort had no measurable effect (Figure 3c, 0.98 vs. 2.92%, respectively, χ^2^[1, *n* = 445] = 1.432, *p* = 0.23). Cell counts used to determine CC3 + Olig2+ frequency are reported in Figure 3d. Thus, DFP reduces OPC survival and this effect is not prevented by Cort in the Cort+DFP condition.

To determine the effects of AChE inhibition and Cort on oligodendrocyte lineage cell maturation, OPCs were differentiated into oligodendrocytes using N1 differentiation media for 3 days in the presence of Cort alone, DFP alone, and in the presence of both Cort and DFP. The data show that Cort (Figure 3e, 33.5 vs. 71.7%, respectively, χ^2^[1, *n* = 453] = 28.962, *p* < 0.001), DFP (Figure 3e, 33.5 vs. 42.9%, respectively, χ^2^ [1, *n* = 611] = 6.371, *p* = 0.012), and Cort+DFP (Figure 3e, 33.5 vs. 55.1%, respectively, χ^2^[1, *n* = 639] = 11.133, *p* = 0.001) conditions increased the number of Olig2+ cells that were post‐mitotic and pre‐myelinating (CC1+). Cell counts used to determine CC1 + Olig2+ frequency are reported in Figure 3f. Cort treatment alone also increased the number of MBP+ mature myelinating oligodendrocytes (Figure 3g, 5.2 vs. 9.9%, respectively, χ^2^[1, *n* = 453] = 8.419, *p* = 0.004). DFP (Figure 3g, 5.2 vs. 7.0%, χ^2^ [1, *n* = 611] = 2.083, *p* = 0.149) and Cort+DFP (Figure 3g, 5.2 vs. 6.7%, respectively, χ^2^[1, *n* = 639] = 2.449, *p* = 0.118) had no significant effect on the number of MBP+ mature oligodendrocytes. Cell counts used to determine MBP+ Olig2+ frequency are reported in Figure 3h.

To interpret whether the effects of DFP were due to cholinergic or non‐cholingeric signaling we measured the levels of ACh in the culture serum. We previously showed that neither OPCs nor astrocytes secrete ACh. We measured no detectable ACh in the media containing 10 or 0.2% FBS used for OPC proliferation and differentiation, respectively, using a fluorometric assay with a sensitivity of ≥100 pmol. Since there was no detectable ACh in either culture medium, the observed responses were due to direct and non‐cholinergic effects of Cort and DFP. Taken together, the data demonstrates that Cort inhibits proliferation and drives maturation of OPCs by direct action on these cells, while DFP increases maturation marginally, but also stimulates apoptosis. In combination with Cort, the toxicity of DFP in vitro acts to counter the pro‐maturation effects of Cort.

To test if ACh alone could directly affect MBP expression, we treated oligodendrocytes with ACh in vitro. We found that elevated ACh was not sufficient to increase MBP expression in cell‐culture (Figure S2a, b, 0.731 ± 0.0995 (vehicle) vs. 0.824 ± 0.105 (treated) MBP/GAPDH ratio, respectively, *t*[2] = 0.816, *p* = 0.55).

### 3.3 AChE inhibition decreases frequency of mature oligodendrocytes in the PFC

**Figure 4:**
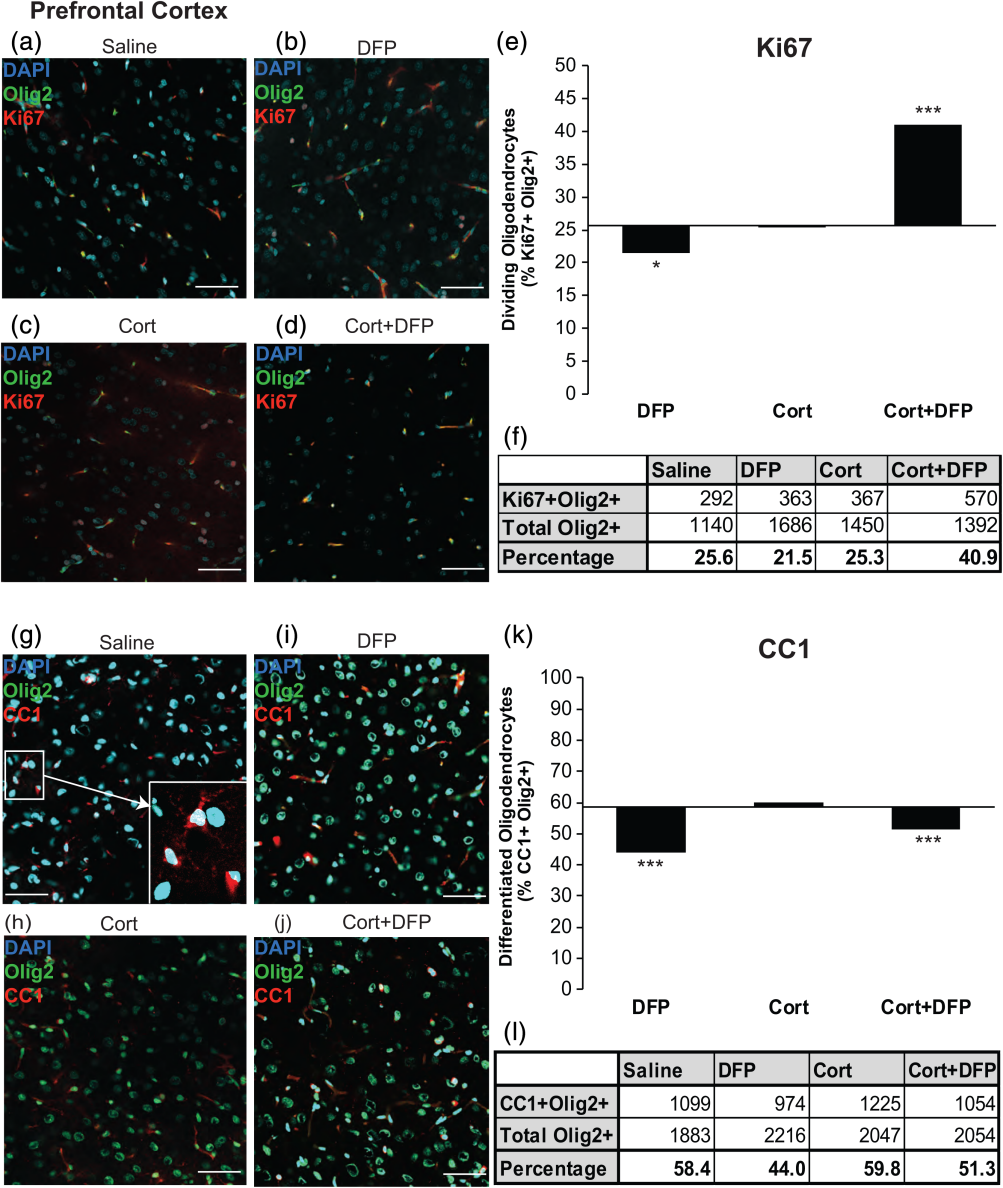
AChE inhibition decreases the frequency of mature oligodendrocytes in the prefrontal cortex (PFC) of the GWI animal model. Data are cell counts from immunohistochemistry of GWI animals at 24 hr postexposure for proliferation analysis and 21 days postexposure for maturation analysis. (a–d) Representative images of Olig2+ Ki67+ cells across treatment conditions. (e) Fraction of proliferating oligodendrocytes (Olig2+ Ki67+) compared to total oligodendrocytes (Olig2+) in the PFC varied with treatment condition (χ^2^ [1, *N* = 3] = 158.86, *p* < 0.00001). DFP decreased the frequency of proliferating oligodendrocytes (χ^2^ [1, *n* = 1686] = 6.3703, *p* = 0.01195). Cort had no effect on Ki67+ oligodendrocyte frequency (χ^2^ [1, *n* = 1450] = 0.031, *p* = 0.8602). Cort+DFP condition had significantly more proliferating oligodendrocytes than saline control (χ^2^ [1, *n* = 1392] = 65.6309, *p* < 0.00001) Bar graphs are total cell counts (*N* = 5, *n* = 50). X‐axis is drawn at the saline control value. (f) Table of proliferating (Ki67+ Olig2+) cell counts in the PFC for each condition. (g–j) Representative images of CC1+ Olig2+ cells across treatment conditions. Inset in (g) illustrates CC1+ (red) Olig2+ (green) and CC1+ Olig2+ (yellow) cell identification. (k) Fraction of mature oligodendrocytes (Olig2+ CC1+) compared to total oligodendrocytes (Olig2+) in the PFC varied with treatment condition (χ^2^ [1, *N* = 3] = 135.04, *p* < 0.001). Bar graphs are total cell counts (*N* = 3, *n* = 30). X‐axis is drawn at the saline control value. DFP treatment resulted in significantly fewer mature oligodendrocytes than saline condition. (χ^2^ [1, *n* = 2216] = 27.611, *p* < 0.001). Cort alone had no effect on CC1+ oligodendrocytes in the PFC (χ^2^ [1, *n* = 2047] = 0.888, *p* = 0.346). Cort+DFP was associated with significantly fewer mature oligodendrocytes than saline condition (χ^2^ [1, *n* = 2054] = 5.7633, *p* = 0.0163). (l) Table of mature (Olig2+ CC1+) cell counts in the PFC for each condition. Scale bar on all representative images is 40 μM. * indicates *p* < 0.05, *** indicates *p* < 0.001, significance is determined by comparing all treatment conditions with the control condition, saline

Cell culture studies indicate that Cort and DFP have complex effects on OPC proliferation, survival, and maturation. Oligodendrocytes in vivo are likely affected by these agents, making it important to determine how oligodendrocytes may be affected in the GWI animal model. Any differences found in the animal model from the results in cell culture will indicate systemic effects and neuron–glia interactions that are not modeled in culture. In addition, the exposures in vivo could be different due to metabolic breakdown of the compounds. We examined the expression of CC1, a nuclear marker for postmitotic but pre‐myelinating oligodendrocytes (Bin, Harris, & Kennedy, 2016), in the PFC of GWI rat model brains at 21 days post treatment with DFP. The PFC was chosen for analysis because this brain region is implicated in GWI and the PFC is still undergoing myelination in humans in the third decade of life (Miller et al., 2012). Representative immunohistochemistry images from the PFC used for quantification are reported in Figure 4a–d. Exposure to the AChE inhibitor, DFP, and Cort had different effects on oligodendrocyte cell proliferation, with DFP being inhibitory (Figure 4b, e, f, 25.6 vs. 21.5%, respectively, χ^2^[1, *n* = 1686] = 6.370, *p* = 0.0119) and Cort having no significant effect (Figure 4c, e, f, 25.6 vs. 25.3%, respectively, χ^2^[1, *n* = 1450] = 0.031, *p* = 0.860). Paradoxically, in combination, DFP + Cort increased the number of proliferating oligodendrocytes (Figure 4d–f, 25.65 vs. 40.9%, respectively, χ^2^[1, *n* = 1392] = 65.6309, *p* < 0.00001). Cell counts of frequencies of proliferating oligodendrocytes (Ki67+ Olig2+) are reported in Figure 4f. In terms of maturation in the PFC, DFP also inhibited maturation to the CC1+ stage, (Figure 4i, k, l, 58.4 vs. 44.0%, respectively, χ^2^[1, *n* = 2216] = 27.611, *p* < 0.001) and Cort with DFP (Figure 4j–l, 58.4 vs. 51.3%, respectively, χ^2^[1, *n* = 2054] = 5.763, *p* = 0.0163) had a similar effect, but Cort treatment alone did not affect maturation of oligodendrocytes to the CC1+ stage (Figure 4h, k, l, 58.4% vs. 59.8%, respectively, χ^2^ [1, *n* = 2047] = 0.888, *p* = 0.346). Representative immunohistochemistry images from the PFCare shown in Figure 4g–j. Tables of cell counts of mature oligodendrocytes (Olig2+ CC1+) for the PFC are reported in Figure 4l.

### 3.4 Effects of GW agents on oligodendrocytes in corpus callosum

**Figure 5:**
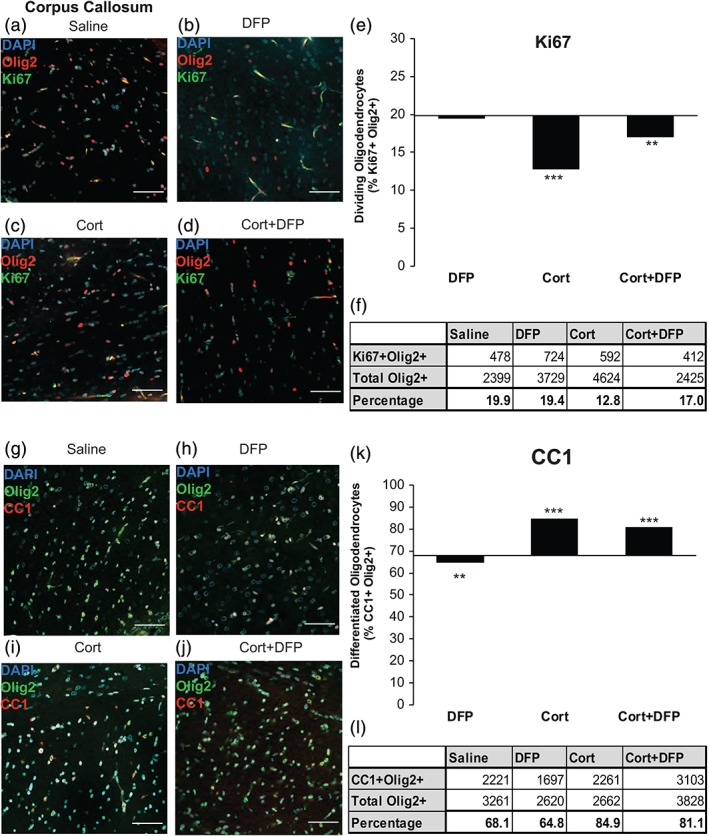
Corticosterone increases the frequency of mature oligodendrocytes in the corpus callosum of the GWI animal model. Data are cell counts from immunohistochemistry of GWI animals at 24 hr postexposure for proliferation analysis and 21 days postexposure for maturation analysis. (a–d) Representative images of Ki67+ Olig2+ cells across treatment conditions. (e) Fraction of proliferating oligodendrocytes (Olig2+ Ki67+) compared to total oligodendrocytes (Olig2+) in the corpus callosum was dependent on treatment condition (χ^2^ = 88.142, *p* < 0.001). Bar graphs are total cell counts (*N* = 5, *n* = 50). X‐axis is drawn at the saline control value. DFP had no effect on the frequency of proliferating oligodendrocytes in the corpus callosum (χ^2^ [1, *n* = 3,729] = 0.240, *p* = 0.624). Cort treatment was associated with significantly fewer proliferating oligodendrocytes (χ^2^ [1, *n* = 4,624] = 60.080, *p* < 0.001). Cort+DFP treatment was associated with significantly fewer proliferating cells than saline condition (χ^2^ [1, *n* = 2425] = 6.910, *p* = 0.009). (f) Table of mature (Olig2+ CC1+) cell counts in the corpus callosum for each condition. (g–j) Representative images of Olig2+ CC1+ cells across treatment conditions. (k) Fraction of mature oligodendrocytes (Olig2+ CC1+) compared to total oligodendrocytes (Olig2+) in the corpus callosum was dependent on treatment condition (χ^2^ = 444.328, *p* < 0.001). Bar graphs are total cell counts (*N* = 5, *n* = 50). X‐axis is drawn at the saline control value. Cort treatment resulted in significantly more mature oligodendrocytes than saline condition. (χ^2^ [1, *n* = 2,662] = 225.445, *p* < 0.001). Cort+DFP was also associated with significantly more mature oligodendrocytes than saline condition, (χ^2^ [1, *n* = 3,828] = 157.995, *p* < 0.001). DFP alone had significantly fewer mature oligodendrocytes (χ^2^ [1, *n* = 2,620 = 7.275, *p* = 0.007). (l) Table of mature (Olig2+ CC1+) cell counts in the corpus callosum for each condition. Scale bar on all representative images is 40 μM. ** indicates *p* < 0.01; *** indicates *p* < 0.001, significance is determined by comparing all treatment conditions with the control condition, saline

The PFC is implicated in GWI and it is still undergoing myelinating in young adults. The major white matter tract in rodents is the corpus callosum. We therefore analyzed the effects of GW agents on oligodendrocyte lineage cells residing in grey matter PFC versus white matter corpus callosum. The effects of DFP and Cort on proliferation and maturation of OPCs differed in the corpus callosum compared with the PFC. This is not unexpected, given the differences in the cellular composition and environment in these two regions. In the corpus callosum, 19.9% of Olig2+ cells were proliferating, immature OPCs, but in the PFC more cells were in this state (25.6%), as this grey matter region is still undergoing active myelination. In the corpus callosum, Cort (Figure 5c, e, f, 19.9 vs. 12.8%, respectively, χ^2^ [1, *n* = 4624] = 60.080, *p* < 0.001), but not DFP (Figure 5b, e, f, 19.9 vs. 19.4%, respectively, χ^2^[1, *n* = 3729] = 0.240, *p* = 0.624) decreased OPC proliferation and this effect persisted when Cort was delivered together with DFP (Figure 5d–f, 19.9 vs. 17.0%, respectively, χ^2^[1, *n* = 2425] = 6.910, *p* = 0.009). Cell counts for corpus callosum oligodendrocyte proliferation frequencies are reported in Figure 5f. In the corpus callosum, differentiation to the CC1+ stage was also promoted by Cort (Figure 5i, k, l, 68.1 vs. 84.9%, respectively, χ^2^[1, *n* = 2662] = 225.445, *p* < 0.001) and Cort + DFP (Figure 5j, k, l, 68.1 vs. 81.1%, respectively, χ^2^[1, *n* = 3828] = 157.995, p < 0.001), but DFP alone decreased maturation (Figure 5h, k, l, 68.1 vs. 64.8%, respectively, χ^2^(1, *n* = 2620 = 7.275, *p* = 0.007). Thus, Cort has opposite effects in the PFC and corpus callosum on proliferation and maturation. DFP decreased proliferation and differentiation in both regions, but the effects in the corpus callosum are either small or not statistically significant.

### 3.5 GWI animals have increased myelin basic protein levels in subcortical white matter

**Figure 6:**
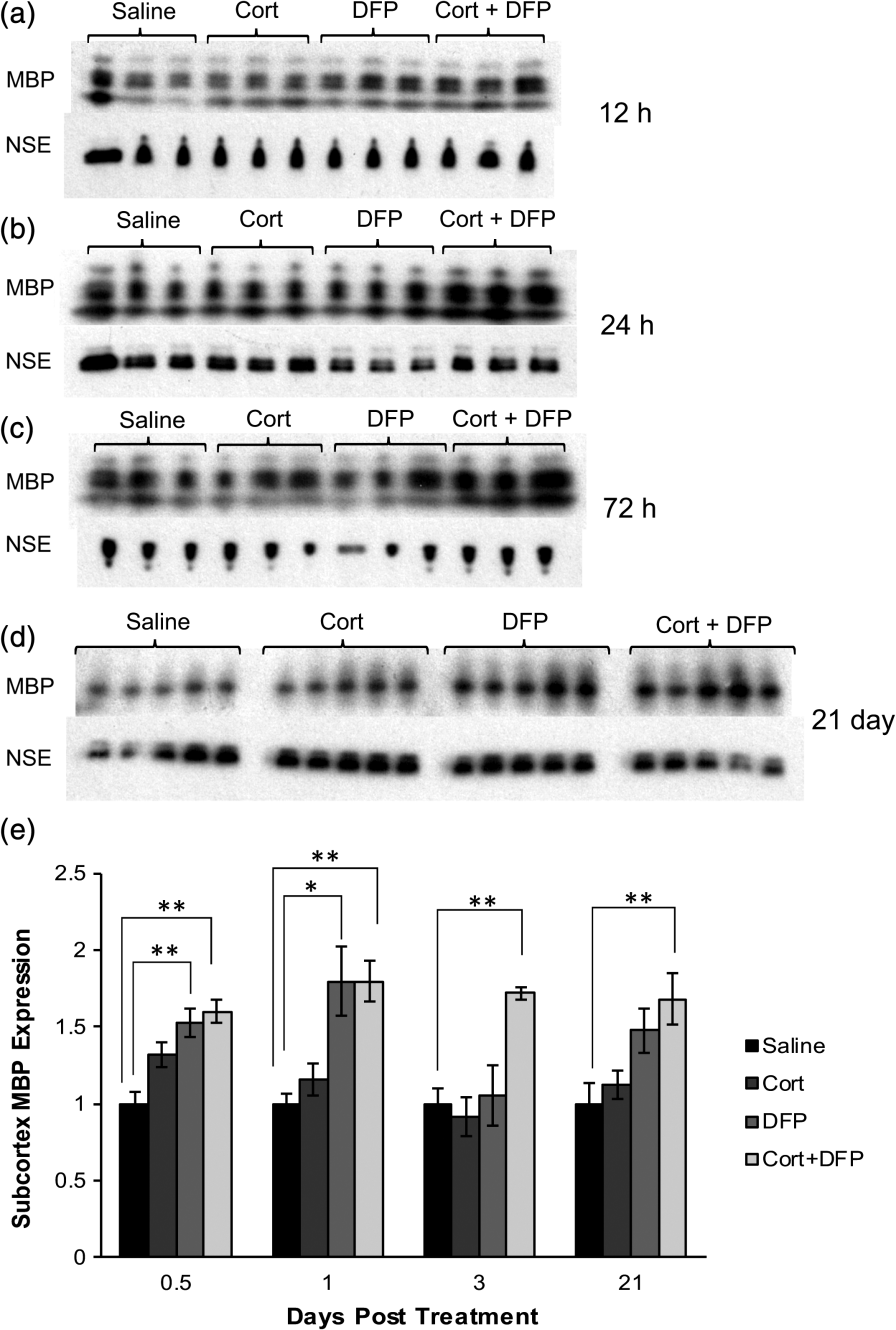
GWI treatment paradigm increases levels of myelin basic protein (MBP) in subcortical white matter. Immunoblots of subcortical white matter homogenates indicate MBP levels after treatment with saline, Cort, DFP, and Cort+DFP at (a) 12 hr, (b) 24 hr, (c) 72 hr, and (d) 21 days posttreatment. MBP isoforms correspond to four immunoblot bands with molecular weights of 21, 18, 17, and 14 kDa. NSE band occurs at 47 kDa. (e) MBP expression was quantified with densitometry and normalized to neuron‐specific enolase (NSE). Each treatment was compared to the control condition, saline, at each time point (12 hr: ANOVA *F*[3, 8] = 10.97, *p* = 0.0033; 24 hr: ANOVA *F*[*3*,8] = 11.19, *p* = 0.0031; 72 hr: ANOVA *F*(*3*,8) = 8.81, *p* = 0.0065; 21 days: ANOVA *F*(*3*,16) = 5.258, *p* = 0.0102. Dunnett's multiple comparison: Saline vs. DFP. *p* < 0.01; saline vs. Cort+DFP, *p* < 0.01). Cort+DFP cotreatment significantly increased MBP levels beginning at 12 hr and persisting at 1, 3, and 21 days postexposure (Dunnett's multiple comparison: Saline vs. Cort+DFP, *p* < 0.01 each time point). Data are reported from *N* = 3 or 5 animals per condition. * indicates *p* < 0.05, ** indicates *p* < 0.01

Changes in the integrity of white matter tracts has been found to underlie the key cognitive and sensory impairments in GW veterans (Rayhan et al., 2013; Van Riper et al., 2017). Therefore, we sought to understand next how the changes in oligodendrocyte development observed in the corpus callosum of the GWI animal model translates to myelin formation. By using immunoblot to measure myelin basic protein (MBP) levels in subcortical white matter, we found that GWI animals co‐treated with Cort and DFP had significantly increased levels of MBP in subcortical white matter as early as 12 hr posttreatment (Figure 6a, e, 1.0 ± 0.071 vs. 1.600 ± 0.078 relative MBP expression, respectively, ANOVA *F*[3, 8] = 10.97, *p* = 0.0033, Dunnett's post hoc analysis: saline vs. Cort+DFP *p* < 0.01) and this effect persists at 24 hr (Figure 6b, e, 1.0 ± 0.070 vs. 2.015 ± 0.134 relative MBP expression, respectively, ANOVA *F*[3, 8] = 11.16, *p* = 0.0031, Dunnett's post hoc analysis: saline vs. Cort+DFP *p* < 0.01), 72 hr (Figure 6c, e, 1.0 ± 0.103 vs. 1.7 ± 0.0384 relative MBP expression, respectively, ANOVA *F*[3, 8] = 8.81, *p* = 0.0065, Dunnett's post hoc analysis: saline vs. Cort+DFP *p* < 0.01) and 21 days (Figure 6d, e, 1.0 ± 0.136 vs. 1.67 ± 0.165 relative MBP expression, respectively, ANOVA *F*[3, 16] = 5.258, *p* = 0.0102, Dunnett's post hoc analysis: saline vs. Cort+DFP *p* < 0.01). Subcortical white matter was the ideal medium for the immunoblot experiments because it is enriched in white matter, including the corpus callosum, which allowed for robust testing of the hypothesis. For the histology experiments, we chose a specific area, the corpus callosum, to eliminate heterogeneity of each subcortical region.

Interestingly, DFP treatment alone produced a significant increase in MBP expression at 12 hours (Figure 6a, e, 1.0 ± 0.071 vs. 1.522 ± 0.0928 relative MBP expression, respectively, Dunnett's multiple comparison: saline vs. DFP, 12 hours: p < 0.01) and 24 hours (Figure 6b, e, 1.0 ± 0.070 vs. 1.795 ± 0.226 relative MBP expression, respectively, Dunnett's multiple comparison: saline vs. DFP *p* < 0.05). Histological analysis showed that at later time points, Cort and Cort + DFP increased the number of mature oligodendrocytes in the corpus callosum (Figure 5k), which is consistent with increased subcortical MBP levels measured by immunoblot. The observed changes in protein expression in vivo were detected as early as 12 hr following treatment, which is too early to be caused by changes in OPC proliferation and differentiation, as the cell cycle of OPCs is ~25–30 hr (Durand, Gao, & Raff, 1997). Thus, the early increase in MBP protein must reflect an increase in its synthesis. Immunoblot analysis of a marker of OPCs supports this conclusion. We measured OPC membrane protein marker NG2 by immunoblot analysis after 12, 24, and 72 hr (Figure S3a–d) and abundance of the transcription factor Olig2 at 12 and 24 hr (Figure S3e–g). GW agents significantly altered NG2 levels 12 hr postexposure but post‐hoc analysis revealed no significant pairwise difference between saline and individual GW agents treatment conditions (Figure S3a,d, 1.0 ± 0.1369 vs. 0.643 ± 0.1788 vs. 1.09 ± 0.211 vs. 1.95 ± 0.365, ANOVA *F*[3, 8] = 5.398, *p* = 0.0252, Dunnett's multiple comparisons, saline vs. Cort *p* > 0.05; saline vs. DFP *p* > 0.05; saline vs. Cort+DFP *p* > 0.05). At 1 day postexposure GW agents significantly altered NG2 levels (Figure S3b, d, 1.0 ± 0.335 vs. 0.816 ± 0.0738 vs. 1.84 ± 0.0734 vs. 1.226 ± 0.174 relative NG2 levels, ANOVA F[3, 8] = 5.2, *p* = 0.0277) and post‐hoc analysis reveals DFP treatment significantly increased NG2 levels compared to saline (1.0 ± 0.335 vs. 1.226 ± 0.174, respectively, Dunnett's multiple comparisons, saline vs. Cort+DFP *p* < 0.05). This could imply an early spurt in proliferation due to increased availability of ACh, which promotes proliferation, via DFP mediated inhibition of AChE, however NG2 is expressed by perivascular and other cells (Smyth et al., 2018), and the amount of protein can be regulated without changes in number of types of cells. At 3‐days postexposure (Figure S3c, d, 1.0 ± 0.0859 vs. 0.676 ± 0.0545 vs. 1.073 ± 0.056 vs. 0.994 ± 0.176 relative NG2 levels, ANOVA *F*[3, 8] = 2.825, *p* = 0.1068), there were no significant differences between the treatment conditions, suggesting an increase in the proliferative pool of OPCs was likely not the sole source of the persistently increased MBP across early and later time‐points. Abundance of transcription factor Olig2 at 12 hr (Figure S3e, g, 1.0 ± 0.283 vs. 0.965 ± 0.158 vs. 0.790 ± 0.0430 vs. 0.460 ± 0.05 relative Olig2 levels, ANOVA *F*[3,4] = 1.48, *p* = 0.3472) and 24 hr (Figure S3f, g, 1.0 ± 0.122 vs. 0.8255 ± 0.170 vs. 1.6876 ± 0.326 vs. 1.365 ± 0.0256 relative Olig2 levels, ANOVA *F*[3,4] = 2.618, *p* = 0.1877) was not significantly different among any treatment condition at these early timepoints, consistent with the transient changes in NG2 levels resulting from factors other than the number of oligodendrocytes.

Alternatively, neuroinflammation and injury can alter axonal sprouting (Chen & Zheng, 2014), which would create new axonal branches to be myelinated, thus stimulating synthesis of MBP. To test this hypothesis, we measured the levels of growth associated protein 43 (GAP43), a marker for axonal sprouting (Benowitz & Routtenberg, 1997) and found that GAP43 levels in subcortical white matter did not change after any treatment condition at 12 hr (Figure S4a, d, 1.0 ± 0.0126 vs. 1.224 ± 0.126 vs. 1.169 ± 0.131 vs. 0.877 ± 0.114 relative GAP43 levels, ANOVA *F*[3,8] = 2.179, *p* = 0.1684), 24 hr (Figure S4b, d, 1.0 ± 0.017 vs. 2.249 ± 0.6929 vs. 2.342 ± 0.354 vs. 0.885 ± 0.263 relative GAP43 levels, ANOVA *F*[3,8] = 3.638, *p* = 0.0640), or 72 hr (Figure S4c, d, 1.0 ± 0.299 vs. 0.674 ± 0.152 vs. 0.807 ± 0.0355 vs. 0.472 ± 0.123 relative GAP43 levels, ANOVA *F*[3,8] = 1.534, *p* = 0.2790) postexposure to DFP and Cort, either alone or in combination. Therefore, the increase in MBP is not explained by myelination of new axons.

Additionally, to test if changes in astrocyte biology were responsible for increased subcortical MBP levels, we measured the levels of the astrocytic protein, GFAP, by immunoblot. GFAP levels remained unaffected by treatment conditions at 12 hr (Figure S5a, d, 1.0 ± 0.200 vs. 0.920 ± 0.080 vs. 1.094 ± 0.059 vs. 0.815 ± 0.064 relative GFAP levels, ANOVA *F*[3,8] = 1.039, *p* = 0.426), 24 hr (Figure S5b, d, 1.0 ± 0.187 vs. 1.142 ± 0.025 vs. 1.53 ± 0.149 vs. 1.383 ± 0.151 relative GFAP levels, ANOVA *F*[3,8] = 2.826, *p* = 0.1067) and 72 hr (Figure 5c,d, 1.0 ± 0.044 vs. 1.385 ± 0.216 vs. 1.525 ± 0.09 vs. 1.23 ± 0.150 relative GFAP levels, ANOVA *F*[3,8] = 2.543, *p* = 0.1295) posttreatment. This is consistent with studies showing GFAP expression is not altered up to 3 days posttreatment with GW agents (O'Callaghan et al., 2015). However, at 21 days post treatment, GW agents significantly affected GFAP expression (Figure S5d, e, 1.0 ± 0.0877 vs. 0.697 ± 0.066 vs. 0.627 ± 0.0417 vs. 1.040 ± 0.113 relative GFAP levels, ANOVA *F*[3,16] = 6.638, *p* = 0.0040). GFAP expression was significantly reduced with Cort alone (Dunnett's multiple comparisons: saline vs. Cort, 1.0 ± 0.0877 vs. 0.697 ± 0.066, respectively, *p* < 0.05) and DFP alone (Dunnett's multiple comparisons: saline vs. DFP, 1.0 ± 0.0877 vs. 0.627 ± 0.0417, respectively, *p* < 0.05) treatment conditions. These observations are consistent with decrease in GFAP expression with chronic exposure to Cort (O'Callaghan et al., 1991; Nichols, Osterburg, Masters, Millar, & Finch, 1990) and DFP (Gupta & Abou‐Donia, 1995) reported in other studies.

The results do not support axonal sprouting, early OPC proliferation or gliosis as the source of increased MBP. In conclusion, immunoblot of subcortical white matter indicates persistently elevated MBP protein levels in subcortical white matter, potentially via elevated MBP protein production at early timepoints (i.e., 24 hr) and by an increase in the number of mature oligodendrocytes in the corpus callosum at later timepoints (21 days). Further, the finding that exogenous ACh on an in vitro monoculture of OPCs is insufficient to change MBP protein levels (Figure S2) suggests the necessity of interactions with axons as axons provide physical support and an appropriate substrate for myelination to promote MBP production in oligodendrocytes (Wake et al., 2011).

Together these results show effects on OPC proliferation, survival, maturation, and increased myelin basic protein expression in the subcortical white matter resulting from treatment with Cort and DFP in the GWI animal model. These effects differ in different brain regions and in cell culture because of the differential contribution of cholinergic and non‐cholinergic effects of GW agents and differences in cellular environments of grey and white matter regions where these oligodendroglial populations reside (Table 2).

**Table 2:** Summary of the effects of GW agents on development of oligodendrocyte lineage cells based on drug treatments and experimental methods

A.					
		Cort			
		Sample			
		In vitro	PFC	CC	Subcortical WM
**Developmental marker**	Ki67	↓	∅	↓	X
	CC3	∅	X	X	X
	CC1	↑	∅	↑	X
	MBP	↑	X	X	∅

B.					
		DFP			
		Sample			
		In vitro	PFC	CC	Subcortical WM
**Developmental marker**	Ki67	∅	↓	∅	X
	CC3	↑	X	X	X
	CC1	↑	↓	↓	X
	MBP	∅	X	X	∅

C.					
		CORT+DFP			
		Sample			
		In vitro	PFC	CC	Subcortical WM
**Developmental marker**	Ki67	↓	↑	↓	X
	CC3	↑	X	X	X
	CC1	↑	↓	↑	X
	MBP	∅	X	X	↑

*Note*: Effects of (A) Cort alone, (B) DFP alone, or (C) Cort+DFP on developmental markers for oligodendrocyte lineage cells. in vitro category is data obtained from OPC monoculture. PFC and CC indicate data from cell counts from prefrontal cortex (PFC) and corpus callosum (CC) respectively. Subcortical WM indicates data from immunoblots of subcortical white matter. Up arrows (↑) indicate increase and down arrows (↓) indicate decrease in cell frequency or protein abundance. Null sign (∅) corresponds to no significant effects, while “X” denotes experiments that were not preformed.

## 4 DISCUSSION

In this study we have investigated the plasticity of myelinating glia in the context of white matter abnormalities in GWI and demonstrated that biology and development of oligodendrocyte lineage cells are significantly affected by exposure to AChE inhibiting agents and the stress hormone, corticosterone. We conclude that DFP, a sarin nerve gas surrogate, decreases maturation of OPCs when acetylcholine signaling is present. DFP also decreases proliferation of oligodendrocytes in the PFC, a region with a higher percentage of proliferating oligodendrocytes than in the corpus callosum.

Our finding that DFP decreases maturation of oligodendrocytes is consistent with the current literature about the role of ACh signaling on oligodendrocyte development (Fields et al., 2017). In MS human clinical trials and MS animal models, inhibition of muscarinic receptors and consequent inhibition of ACh signaling is shown to promote remyelination (Abiraman et al., 2015; Green et al., 2017; Li et al., 2015; Liu et al., 2016; Mei et al., 2014; Welliver et al., 2018). Therefore, our finding that elevated ACh signaling decreases the number of mature oligodendrocytes is consistent with the literature. In our non‐cholinergic in vitro studies, DFP increased the frequency of mature oligodendrocytes and also significantly increased apoptosis in OPCs. In previous in vitro studies, organophosphates preferentially affected the maturation and survival of immature neuronal cells (Monnet‐Tschudi, Zurich, Schilter, Costa, & Honegger, 2000). DFP has various non‐cholinergic effects, including neurotoxicity (Qian et al., 2007), immunogenicity (Chaubey et al., 2019), and disruption of axonal transport (Naughton et al., 2018; Rao et al., 2017).

Another factor to consider, is that the CDC GWI animal model includes chronic Cort exposure prior to DFP exposure. In terms of oligodendrocyte and white matter biology, we find that Cort alone has robust protective effects by promoting maturation of oligodendrocytes and decreasing proliferation. These findings are consistent with previous literature exploring the effects of Cort on oligodendrocyte biology (Alonso, 2000; Miyata et al., 2016).

Given the somewhat antagonistic interactions of DFP and Cort, we find that the Cort+DFP condition reveal region specific effects on oligodendrocyte biology. With Cort+DFP treatment, we find that in the PFC, OPCs are pushed into a more proliferative and less mature state. In contrast, with the same treatment paradigm in the corpus callosum, OPCs are pushed to a less proliferative and more mature state. This could be due to the relatively more mature population of oligodendrocytes in the corpus callosum as well as differences in ACh availability in the two regions. The importance of regional differences is highlighted by the reported abundance of cognitive rather than sensory symptoms in the younger GW veterans (Gopinath et al., 2012), implicating the involvement of PFC, a region where myelination continues into the third decade of life (Miller et al., 2012). The decrease in mature oligodendrocytes identified in the PFC suggests that the PFC is especially vulnerable to GW agents; a finding that is consistent with the neuropsychological impairments presented by GW veterans (Janulewicz et al., 2017; Sullivan et al., 2003; Sullivan et al., 2018). Our cell‐culture studies more closely match the data from the corpus callosum where Cort+DFP promotes maturation of oligodendrocytes. Given that there is no detectable ACh in our OPC monocultures, we find that promotion of maturation by Cort overwhelms the anticholinesterase effect of DFP.

MBP protein was elevated in subcortical white matter. The increase in MBP levels with Cort+DFP co‐treatment, could be due to promotion of the oligodendrocyte lineage toward a more mature state by Cort in the corpus callosum. However, an increase in MBP does not necessarily imply a healthier and more functional white matter (Kristensson et al., 1986). Increased MBP mRNA transcripts have been reported during periods of demyelination, providing evidence for a compensatory increase in MBP levels in response to pathology (Kristensson et al., 1986). A transient increase in MBP mRNA, occurring within 6 hr, has been previously reported as an oligodendroglial cellular response to injury (Bartholdi & Schwab, 1998). This argument is underscored by studies showing that DFP exposure of rats increases myelin decompaction while having no effect on the g‐ratio or white matter volume (Naughton et al., 2018). Therefore, it is important to note the complicated relationship between MBP levels, oligodendrocyte maturity, and myelin integrity.

The results of the quantitative histological analysis of oligodendroglia in the PFC of the GWI rat model is consistent with RNA‐seq data in mice showing that combined Cort and DFP treatment decreases the fraction of mRNA transcripts associated with mature oligodendrocytes in the PFC (Ashbrook et al., 2018), but our results are not consistent with this gene profiling study in other respects. Cort treatment alone did not alter the number of mature oligodendrocytes in the PFC, but mRNA transcripts associated with oligodendrocytes are reportedly reduced in this condition (Ashbrook et al., 2018). Also, the frequency of mature oligodendrocytes in the PFC decreased in response to DFP treatment, but mRNA transcripts associated with mature oligodendrocytes remained unchanged under this condition (Ashbrook et al., 2018). These discrepancies may be explained by the fact that gene and protein abundance are indirect indicators of cell numbers, and gene expression and protein levels are influenced by physiological conditions. Alternatively, methodological or species differences could also account for discrepancies between mRNA profiling and histological analysis.

Based on our findings we predict that GW veterans would have decreased white matter integrity varying by brain region. These predictions are supported by the brain imaging studies of GW veterans (Bierer et al., 2015; Rayhan et al., 2013; Van Riper et al., 2017). The published neuroimaging data reflect both increased and decreased myelin integrity depending on the myelin track analyzed and methodological differences in measurement. For example, previous GWI imaging data with MRI has shown that axial diffusivity in the right inferior fronto‐occipital fasciculus, a white matter tract that links cortical regions involved in fatigue, pain, emotional and reward processing, and the right ventral attention network in cognition, is significantly increased in GW veterans and correlate with the severity of pain and fatigue (Rayhan et al., 2013). In veterans with post‐traumatic stress disorder (PTSD), increased structural integrity has been reported in the cingulum bundle, a white matter tract connecting the right amygdala and anterior cingulate cortex (Bierer et al., 2015). Importantly, an equal number of studies have identified decreased myelin integrity depending on the brain region. It has been shown in veterans with GWI and chronic pain that there is a lower white matter integrity across multiple brain regions including the frontal gyrus, corpus callosum, and precentral gyrus (Van Riper et al., 2017). GW veterans with PTSD also display significantly reduced mean diffusivity in the right, but not left cingulum (Bierer et al., 2015). Our studies show that DFP treatment of OPC monoculture also promotes toxicity. Taken together, the data and available evidence suggest that the effects of AChE inhibition, corticosterone exposure, and their combined treatment, on oligodendrocyte biology and white matter vary depending on the brain region and cell environment, reflecting underlying differences in ACh availability and cellular composition between regions.

## 5 CONCLUSION

Our study shows that impairment of oligodendrocyte biology is an important aspect of the pathophysiology of GWI. We have identified that DFP, an analog to sarin nerve gas, reduces the frequency of differentiated oligodendrocytes across multiple brain regions. Our data shows that Cort, used in the CDC animal model, antagonizes the effects of DFP, as Cort alone increases the frequency of differentiated oligodendrocytes. With co‐treatment of Cort and DFP, we find a lower frequency of CC1+ oligodendrocytes in the PFC and higher frequency of CC1+ oligodendrocytes in the corpus callosum. The cell count data in the corpus callosum is supplemented and corroborated by elevated MBP levels in the sub cortex. These differences highlight the heterogenous responses of oligodendrocytes to agents implicated in GWI and used in the GWI animal model. Similar heterogeneity is reflected in brain imaging studies and in the wide range of symptoms experienced in GWI. Taken together, these findings suggest therapeutic avenues where restoring the endogenous cholinergic signaling required for normal oligodendrocyte cell biology and function may potentially alleviate the chronic symptoms of veterans with GWI. This study also suggests that civilian exposure to AChE inhibitors, such as commercial pesticides, may have chronic effects on white matter, especially during childhood and early adolescence, when the brain is at its most plastic.

## DISCLAIMER

The findings and conclusions in this report are those of the authors and do not necessarily represent the official position of the National Institute for Occupational Safety and Health, Centers for Disease Control and Prevention, Henry M. Jackson Foundation for the Advancement of Military Medicine, and U.S. Department of Defense.

## Supporting information


**Supplemental Figure 1 Corticosterone and DFP dose–response curves for in vitro OPC monocultures.** (a) Total Olig2 counts (Olig2 + DAPI+) summed over 10 fields of view. DFP doses used were: 0, 1, 5, 10, 50, 100 μM. (N = 3, n = 10 per condition). (b) Total Olig2 counts (Olig2 + DAPI+) summed over 10 fields of view. Corticosterone doses used were: 0, 0.1, 0.5, 1, 5, 10, 50 μM. (N = 3, n = 10 per condition). Bars are average counts per field ± SEM.

**Supplemental Figure 2 ACh treatment alone is not sufficient to induce MBP changes in OPCs** (a) Immunoblots of MBP and GAPDH from differentiating oligodendrocytes with ACh (1 μM in N1 differentiation media) and without ACh (Veh, differentiating media). (b) Immunoblots were quantified with Image J as the ratio MBP/GAPDH (N = 3, Vehicle = 0.731 ± 0.099, ACh = 0.824 ± 0.105, *t(*2) = 0.816, *p* = 0.55). Individual MBP isoform bands were summed to give a single MBP value for each sample.

**Supplemental Figure 3 No evidence for an early increase in OPC population in the GWI animal model.** Immunoblot of whole brain homogenate for NG2 and NSE loading control at (a) 12 hours, (b) 24 hours, and (c) 72 hours post DFP treatment. (d) All treatment conditions were compared to the control condition, Saline. NG2 levels were significantly increased at 24 hours in DFP condition (ANOVA *F*[3, 8] = 5.2, *p* = 0.0277, Dunnett's multiple comparisons saline vs. DFP p < 0.05). No significant difference in NG2 at 12 hours (ANOVA *F*[3, 8] = 5.398, *p* = 0.0252, Dunnett's multiple comparisons saline vs. all conditions, *p* > 0.05) or 72 hours (ANOVA *F*(3, 8) = 2.825, *p* = 0.1068). NG2 band occurs at 300 kDa and NSE occurs at 47 kDa. Immunoblot of subcortical brain homogenate for Olig2 protein and NSE loading control at (e) 12 hours, and (f) 24 hours post DFP treatment. Olig2 band occurs at 32 kDa, NSE band occurs at 47 kDa. (g) No changes in Olig2 protein expression was observed (n = 3 for all conditions, 12 hours: ANOVA *F(*3,4) = 1.48, *p* = 0.3472; 24 hours: ANOVA *F(*3,4) = 2.618, *p* = 0.1877). All treatment conditions were normalized to the control condition, Saline. * indicates p < 0.05.

**Supplemental Figure 4 No change in GAP43 expression in GWI animal model.** Immunoblots of subcortical brain homogenate showing GAP43 expression in Saline, Cort, DFP, and Cort+DFP conditions at (a) 12 hours, (b) 24 hours, (c) 72 hours. GAP43 band occurs at 43 kDa; NSE band occurs at 47 kDa. (d) GAP43 expression levels were not affected by the treatment conditions (n = 3 all conditions, ANOVA 12 hours: *F(*3,8) = 2.179, *p* = 0.1684; ANOVA 24 hours: *F(*3,8) = 3.638, *p* = 0.0640; ANOVA 72 hours: *F(*3,8) = 1.534, *p* = 0.2790). Protein expression levels were normalized to NSE at each time point and all treatment conditions were compared to the control condition, Saline.

**Supplemental Figure 5 No change in GFAP expression in GWI animal model.** Immunoblots of subcortical brain homogenate showing GFAP expression with Saline, Cort, DFP, and Cort+DFP treatment at (a) 12 hours, (b) 24 hours, (c) 72 hours, and (d) 21 days post treatment. (e) GFAP levels were not affected by any treatment condition at 12, 24 or 72 hours (12 hours: ANOVA *F(3*,8) = 1.039, *p* = 0.4263); 24 hours: ANOVA *F(3*,8) = 2.826, *p* = 0.1067; 72 hours: ANOVA *F(3*,8) = 2.543, *p* = 0.1295; 21 days: ANOVA *F(*3,16) = 6.638, *p* = 0.0040) post treatment. Protein expression levels were normalized to NSE at each time point and all treatment conditions were compared to the control condition, Saline.
